# Norjizak Injection: A Critical Risk for Transmitting Blood-Borne Infectious Diseases

**DOI:** 10.5812/hepatmon.8272

**Published:** 2013-04-06

**Authors:** Zahra Alam Mehrjerdi

**Affiliations:** 1 Iranian National Center for Addiction Studies (INCAS), Tehran University of Medical Sciences, Tehran, IR Iran

**Keywords:** Injection, Blood-Borne Pathogens, Infection

Dear Editor,

Infectious diseases including HIV and viral hepatitis constitute a major health concern with high prevalences among injecting drug users (IDUs) in Iran (1, 2). IDUs that share needles and syringes and practice sexual behaviors are at risk groups for being infected with blood-borne infectious diseases such as HCV (3). The recent increasing use of opioids in Iran has been strongly associated with health-related harms (4) including transmitting HIV, HCV, and HBV infections (5-9). In 2005, a new illicit opioid, named as Norjizak, was introduced in Iran’s illicit drug market and it gained popularity in a short period of time and contributed to transmitting blood-borne infectious diseases. Norjizak which is also written as Norjizac, Norgesic, and Norchizack in Iran is a narcotic drug which is a combination of several opioids with Dexamethason or Benzodiazepines (10). Its main route of administration is injection. Norjizak is also used intramuscularly and/or subcutaneously. Its injection is associated with several medical complications including abscess formation, development of septic emboli and soft tissue infections in IDUs, but a few studies have focused on Norjizak injection and its health-related problems in Iran (11). A recent study on the patterns of pre-treatment drug abuse, drug treatment history, and characteristics of 810 drug dependent-patients at drug use treatment clinics in Tehran, Iran showed that Norjizak injecting was prevalent as a main drug of abuse among 0.5% of the sample before entry to Methadone maintenance treatment (12). In another study on hospitalized drug users in Loghman Hakim and Imam Hussein hospitals in southern Tehran, a total of 221 patients were studied. The study results showed that 5.4% included injecting Norzijak users (INUs) (13). A research study on 18 male Norjizak-dependent patients with a mean age of 29.8 years who volunteered for treatment at a drug use treatment clinic showed various psychological and physical impacts associated with Norjizak injecting such as striae, weakness, high systolic blood pressure, moon face, hirsutism, extensive dermal infection, gynecomastia, backache, insomnia, lack of potency, and 22.2% of the sample was infected with HCV (14). In a study on infectious complications and mortality due to Norjizak injecting in comparison with other injecting opioids, 690 patients whom were admitted to a hospital in Isfahan city were studied. The study findings showed that 14.5% were INUs. Compared with other groups, Norjizak group was younger and the death rate was 37.5% which was higher than other groups (15). In a study, 14 vials of Norjizak were tested for detecting HCV. Two-hundred micro-liters of the solution in each vial were obtained and using a high pure viral nucleic acid kit, RNA extraction was done. Thereafter, C-DNA was produced by the use of Moloney Murine Leukemia Virus reverse transcriptase (Fermentas, Life Science). Using specific primers and a probe for the detection of HCV (Tagman probe), PCR was done (Corrbette Research 6000). The study results showed that 14% of the vials were positive for HCV (16). Norjizak injecting is a crucial health concern and risky behaviors related to norjizak injecting account for a considerable proportion of incident blood-borne infectious diseases such as HCV. Therefore, treatment interventions and harm reduction programs are necessary to prevent or reduce Norjizak injecting among IDUs. With reducting Norjizak injecting and consequently, decreasing the spread of blood-borne infectious diseases, these programs help both INUs and the community. There is evidence that access to Methadone maintenance treatment (MMT) could be helpful for reducing illicit drug injection and can be considered as a major intervention for preventing from transmitting blood-borne infectious diseases in Iran (17). In recent years, a harm reduction approach has been rapidly developed for IDUs in Iran. Methadone and Buprenorphine maintenance treatment programs have become widespread (18). A study on MMT outcomes in Iran showed that the prevalence of drug injection in the MMT group was lower compared with the control group (16% vs. 100%). There was also a considerable difference in needle and syringe sharing (40% in the control group vs. 4% in the MMT group) (19). In recent years, needle and syringe programs (NSPs) have been also developed to decrease sharing practices among IDUs in Iran (20, 21), but implementing specific harm reduction strategies that specifically target INUs should be considered by policymakers and health providers. The medical and health impacts of Norjizak injecting in Iran are complex and devastating, therefore, extensive research is still required to investigate the effects of Norjizak injecting on general health and transmitting blood-borne infectious diseases. Drug education on Norjizak injecting and its risks for being infected with viral infections such as HCV is essential. Norjizak injecting is a critical health concern among some Iranian IDUs that requires a collaborative and consistent response among all health providers. Treatment interventions and harm reduction programs should be specifically implemented for the current treatment needs of INUs in Iran.
